# Nurses and Neighbors

**Published:** 1877-10

**Authors:** Thos. K. Beecher


### NURSES AND NEIGHBORS.

THOS. K. BEECHER.

When a man is sick, his help comes from:—

1. His animal vigor—constitution.
2. His wisdom and self control.
3. His physician's skill.
4. His nurses and neighbors.

Some pious reader may be disappointed that the writer being a clergyman—a teacher of religion—has not put first of all helps, *the help of God.* But the help of God is the strength or inspiration which makes the four helps named above possible. There is no animal vigor, no wisdom, no skill, no ministry of nurses or neighbors except as supplied (with more or less of hindrance and dilution from men) by God. That the sick man and his helpers may work with, and not against, the God of our salvation [health] is the thing for all to aim at.

Passing the first three helps, the present endeavor is to speak of the fourth—*nurses and neighbors.*

When child or man falls sick the natural nurses are the women of the family. For this service women are peculiarly and happily endowed. The kind of help needed by the sick belongs mainly to the department of house-keeping. A clean, well-aired room, a tidy bed, delicate "low diet" preparations served appetizingly, modest decorations, few and odorless flowers, and home-like arrangements of furniture, these and such fall usually into the hands of cheerful women.

As an inspiration and strength for the performance of these duties, woman is more highly endowed than most men with a power of continuous loving, that, for the time, causes the loved one to serve as a satisfying center, around which the world of consciousness arranges itself contentedly. This is the "motherly" quality. In virtue of this, babes are cared for with a cheerful assiduity wholly incomprehensible by us that are men. A father comes in and enjoys playing with the baby for a short time. Very few men can "tend" baby. The play is soon over, and we hear the cry, "here, mamma, take the baby," and mamma takes it.

This motherly quality is part of the nurse's equipment. It is an inspiration that alleviates the drudgery of domestic labor. It brings with it, however, some drawbacks.

A nurse for the sick not infrequently is so wholly wrapped up in her patient that she becomes a damage to the one she would help. The outcry of her love lays a tax on the slender strength of the sick one—she loves him so that she cannot let him alone!

A strong swimmer, when rescuing a drowning friend, has need to hold him off, lest the frantic hug of the drowning one disable both, till they drown together. In some cases of sickness the nurse who is quite willing to be clung to by the patient, unconsciously clings to the patient and exhausts him by the demands of solicitude and love.

"O, I can't lay the dear child down. I must hold him—he's my darling," says a mother who is actually distressing the child by holding it. I have known a dying husband to endure an hour of neck ache rather than pain his loving wife by preferring a pillow. Her love spurred her with desire to be doing something for the sufferer all the time. "You feel easier now, don't you?" "Shan't I do this?" "Shan't I do that?" from morning to night—teasing the feeble patient to make chances for love to DO something.

Love is thus at once a strength and a weakness in a nurse. A strength when it serves as an inspiration for continuous and costly services. A weakness when it so overmasters wife, sister or mother as to make them lose self-control and the wisdom possible to the self-poised only.

—The fact as to a sick person is that, he needs a chance. The high art of nursing is to perceive and quietly satisfy every want of the patient, and if possible conceal the work and postpone all wants of one's own. Disinterested love, a Christ-like love is the inspiration needed by a nurse—"I am willing to spend and be spent for you—use me."

A sick man must be helped *according to his nature* and indications, and not according to the professional necessity of his physician, nor the heart wants of his loving nurses. There lies the patient—be he babe or boy, child or man, matters not. Give him a *chance* to get well. Let the prescriptions fit his case, even if they be unscientific and contrary to the school of practice to which you, dear doctor, belong. And let the nursing even more absolutely fit the case, whether the loving heart can find comfort or not.

—To visiting friends and neighbors, the same truths apply. Visiting should never be done for the gratification of curiosity nor even to satisfy one's love. The neighbor has done full duty when calling at the door he says, "Call on me whenever I can help you," It is a prodigious comfort to a stricken family to know that friends are ready at call. It is often a burden to a family having wide acquaintance, to have them coming in so generously that one fears to grieve them, by saying we need to be let alone.

— I find that I have unintentionally opened up a very large subject for discussion—nor more nor less than this:—How can people thoroughly love other people and yet become a bore to neither? In health, this question calls for delicacy and tact in solving it. In sickness the difficulty is even greater. Let it suffice, however, to have called the matter up. A little thoughtfulness on the part of our helpers in sickness will enable them to love no less, while they learn to do far more toward ensuring a recovery which is too often hindered by the very love which is in agony lest the recovery come not. It is not always true that they who love most are able to do most. Only when the loving heart works with the wise head, are the best results attained.

